# The impact of sleep problems on cerebral aneurysm risk is mediated by hypertension: a mediated Mendelian randomization study

**DOI:** 10.3389/fgene.2024.1434189

**Published:** 2024-10-11

**Authors:** Xiaofei Yan, Hongwu Li

**Affiliations:** ^1^ Department of Pathology, The Quzhou Affiliated Hospital of Wenzhou Medical University, Quzhou People’s Hospital, Quzhou, Zhejiang, China; ^2^ Department of Neurosurgery, The Quzhou Affiliated Hospital of Wenzhou Medical University, Quzhou People’s Hospital, Quzhou, Zhejiang, China

**Keywords:** cerebral aneurysm, sleep problems, risk factors, Mendelian randomization, genetic predisposition

## Abstract

**Introduction:**

Cerebral aneurysm (CA) is a common vascular disease. The risk factors of CA include hypertension, smoking, and a family history of genetic predisposition. Although sleep-related problems have been found to have a strong association with cardiovascular disease, there is a lack of research regarding the causal relationship with cerebral aneurysms.

**Methods:**

In this study, we investigated the causal relationship between four sleep-related problems, including snoring, insomnia, narcolepsy, and napping during the day, and CA using a two-sample Mendelian randomization (MR) analysis. Moreover, the potential confounders before sleep problems and CA were further analyzed by multivariate MR (MVMR).

**Results:**

The causal relationship between insomnia and CA was obtained analytically by means of six MR analyses. There was a strong causal effect relationship between insomnia and CA, which suggests this as a potential risk factor [odds ratio (OR) = 8.35, 95% confidence interval (CI) = 2.422–28.791, *p* = 7.772e-04]. On this basis, hypertension was identified as a mediator between insomnia and CA by MVMR, with a mediating effect of 52.538% (OR = 3.05, 95% CI = 1.549–4.55, *p* = 0.015).

**Conclusion:**

The causal relationship between insomnia and CA was predicted using genetic variance data, and insomnia was found to be a potential risk factor. Furthermore, hypertension is a mediator between insomnia and CA. Therefore, focusing on sleep problems and improving sleep quality may be an active and effective strategy to prevent CA.

## 1 Introduction

The main cause is a focal vascular bulge due to vascular malformation (Di et al., 2012), atherosclerosis, or trauma (Miyamoto et al., 2017), and a rupture in this area can lead to a hemorrhage with severe consequences (Marbacher et al., 2020). Moreover, in most patients, CA lacks obvious signs and symptoms before vascular rupture occurs, and only a small number of patients will have some atypical symptoms, such as dizziness, headache, and blurred vision; however, once rupture occurs, patients will have obvious symptoms, such as dizziness and headache (Yanagawa et al., 2013). After an aneurysm ruptures, a large amount of blood leaks out, which can lead to aneurysmal subarachnoid hemorrhage, which progressively increases the mortality rate of the patient by up to ≥40% within a day or even a week after the rupture (English et al., 2018; Akcil et al., 2018). Therefore, it is particularly important to explore the risk factors for CA growth and rupture in this vascular disease with atypical symptoms and a high mortality rate after its occurrence. Common clinical risk factors include hypertension, smoking, obesity, and genetics (Juvela and Korja, 2017; Juvela et al., 2013; Lacolley et al., 2012). Moreover, a recent Mendelian randomization study indicated that lipid levels affect aneurysms and are a risk factor for their development, and the use of lipid-lowering drugs may prevent and treat aneurysms to some extent (Chen et al., 2021). Therefore, the investigation of the prevention and risk factors of CA is urgently required.

Sleep problems are a health issue that many people currently face and can affect peoples’ physical and mental health (Mazza et al., 2020; Choi et al., 2022). These sleep problems mainly include insomnia, drowsiness, and snoring (Svensson et al., 2012), which are mainly related to biological clock disorders (Italianer et al., 2020). Additionally, long-term sleep problems can lead to endocrine disorders and imbalance (Gerashchenko et al., 2017), and even damage the immune system, affecting the body’s immune response function (Spies and Bringmann, 2018). Previous studies have indicated that sleep problems, especially insomnia, can lead to high blood pressure, diabetes, some cardiovascular diseases (Xu et al., 2020), and even some autoimmune diseases. For example, sleep disorders can lead to the development of colitis, and the duration and efficiency of sleep are also strongly associated with the development and progression of inflammatory bowel disease (Tang et al., 2009; Jarasvaraparn et al., 2019). In addition, obstructive sleep apnea in sleep disorders contributes to the risk of stroke occurrence, especially cerebral hemorrhage (Titova et al., 2022). Current studies indicate sleep problems as a threat to cardiovascular disease; however, further research is required, and there is presently a lack of research regarding the causal relationship between sleep disorders and the risk of CA.

Mendelian randomization (MR) is a type of randomized controlled experiment that uses the mutation data of genes as instrumental variables (IVs) to simulate randomized controlled experiments using the principle of random assignment of alleles between parents and offspring and is used to explore the causal relationship between exposure factors and outcome factors (Johnson et al., 2020; Suzuki et al., 2021). The benefit of this approach is that it minimizes interference from external confounding factors, thereby diminishing the impact of confounding bias on the outcome. Additionally, it facilitates the analysis of the causal relationship between exposure and outcome, as alleles are inherited from parents by offspring, an irreversible process (Prins et al., 2019; Akinkugbe et al., 2016). In this study, we used MR analysis to obtain exposure factors related to sleep problems, such as snoring, insomnia, narcolepsy, and napping during the day, as four exposure factors from the genome-wide association study (GWAS) database. Furthermore, we acquired the dataset for CA as an outcome variable and investigated the causal relationship between them through a two-sample MR analysis to ascertain if sleep problems serve as a risk factor for CA. Building on this, we further analyzed the roles of hypertension, smoking, and obesity as potential mediators and the extent of mediation they contributed, using MVMR models.

## 2 Materials and methods

### 2.1 Study design

**FIGURE 1:**
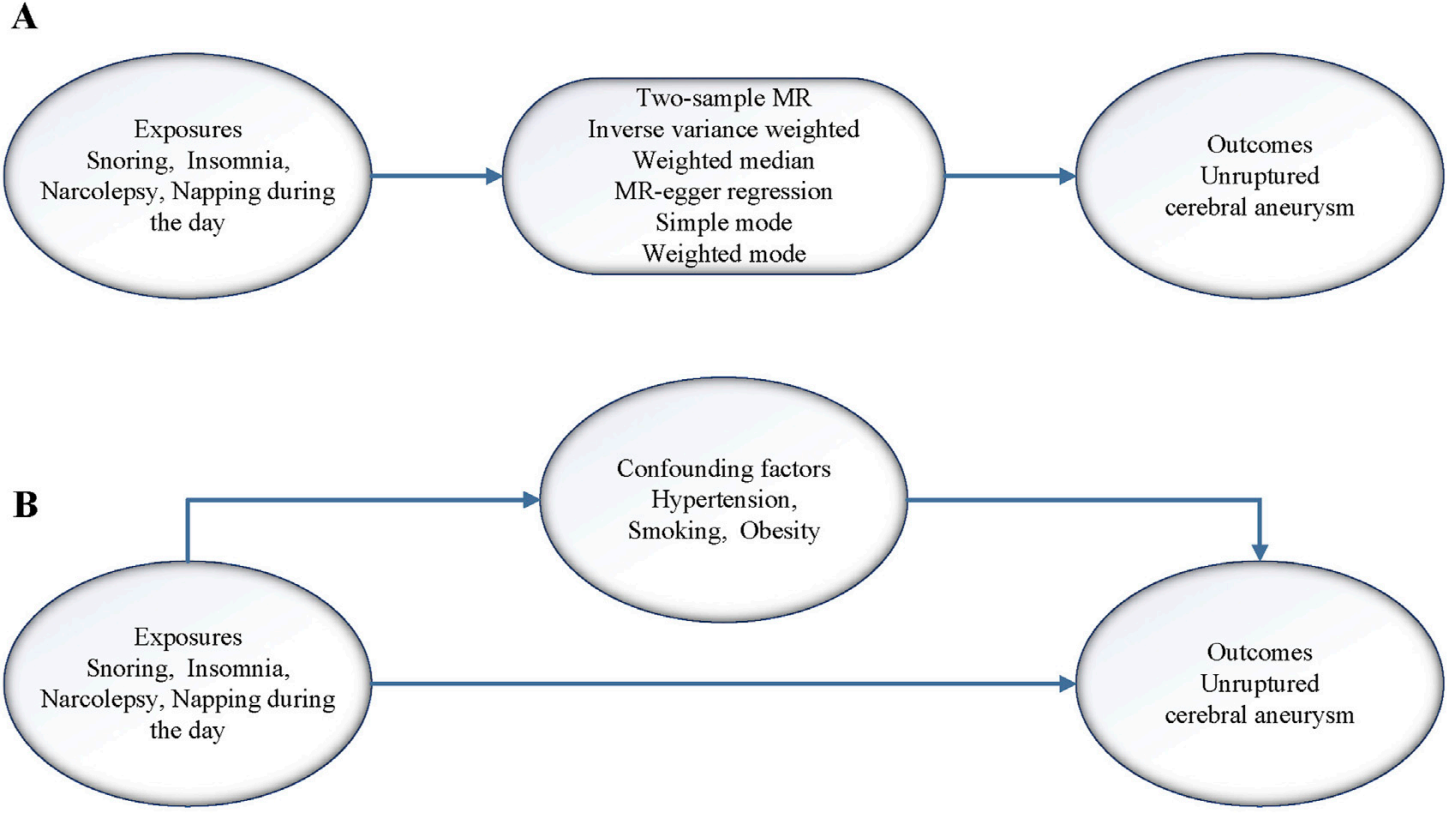
**(A)** Flow of analysis of the two samples MR in this study. **(B)** Flow of analysis of the MVMR in this study.

A flow chart of the design ideas for this study is shown in Figure 1. We first obtained the exposure dataset related to sleep problems from the GWAS database (https://gwas.mrcieu.ac.uk/) and the genetic mutation data from the dataset of CA, which utilized single nucleotide polymorphisms (SNPs) as IVs in this study. Next, we performed heterogeneity and horizontal pleiotropy tests for the screened IVs, and when both tests were met, we performed two-sample and MVMR analyses.

### 2.2 Characteristics of the study population

The genetic data used in this study were obtained from publicly available databases and did not require ethical review. Genetic data for the exposure factors in this study were selected from the UK biobank database, confounders were selected from the Finnish database, and genetic data for the outcome factors were selected from the GWAS database (Sakaue et al., 2021). To fully investigate sleep-related problems, four representative sleep problems were selected, including snoring, insomnia, narcolepsy, and napping during the day. By reviewing the literature, we finally selected hypertension, smoking, and obesity as potential confounders for MR analysis. The outcome data were selected for unruptured CA. These selected dataset features are shown in detail in Table 1. Additionally, to reduce the effect of population bias, our selected datasets were from European ethnic groups. The criteria for SNPs screened as IVs in this study were *r* ^2^ < 0.001 for linkage disequilibrium, a spacing of 10,000 kb or more for linkage disequilibrium, and *p* < 5 × 10^−8^. In addition, to ensure that the IVs could represent the exposure factors, we calculated the F-statistic (F= (*R* ^2^/K)/[(1 –R^2^)(N–K – 1)]) value for validation to test the correlation between them, and an F-statistic value greater than 10 was considered to have a strong correlation, where *R* ^2^ is the coefficient of variance, N is the number of sample cases, and K is the number of IVs.

**TABLE 1:** Characterization of exposure data, mediators, and outcome data.

Exposures	Sample	nSNP	Category or consortium	Population
Snoring	314,449	10,894,596	Neale Lab	European
Insomnia	336,965	10,894,596	Neale Lab	European
Narcolepsy	336,082	10,894,596	Neale Lab	European
Nap during day	337,074	10,894,596	Neale Lab	European
Mediators				
Hypertension	218,754	16,380,466	Binary	European
Smoking	138,088	16,379,853	Binary	European
Obesity	218,735	16,380,465	Binary	European
Outcome				
Unruptured cerebral aneurysm	473,683	24,195,863	PMID: 34594039	European

SNP, single-nucleotide polymorphism; nSNP, number of SNPs.

### 2.3 Sensitivity analyses

The sensitivity analysis in this study was divided into two aspects, including a test of heterogeneity and a test of horizontal pleiotropy. In this study, Q and *p* values of the inverse-variance weighted (IVW) (Burgess et al., 2013) and MR-Egger (Bowden et al., 2015) methods were calculated using Cochrane’s Q test, and horizontal pleiotropy was tested using the MR-Egger intercept and MR-pleiotropy residual sum and outlier (PRESSO) global test. Finally, we performed a separate retention analysis for each SNP using the leave-one-out method to observe its effect on the overall effect.

### 2.4 Two-sample and multivariable MR methods analysis

Based on the results of the heterogeneity and horizontal pleiotropy tests, we next performed a two-sample MR analysis. These six analysis methods include the IVW, weighted median (Bowden et al., 2016), MR-Egger regression, simple mode, weighted mode, and MR-PRESSO (Verbanck et al., 2018) methods. Because the IVW method has a higher statistical efficacy than other methods do, the analysis results of the IVW method were used as the evaluation criteria in this study (Burgess et al., 2017). Additionally, the MR-PRESSO method was used to detect the presence of potential outliers in the IVs and evaluate the situation of pleiotropy. Next, we analyzed whether the potential confounders could serve as mediators in this study through MVMR (Sanderson et al., 2019) separately.

### 2.5 Analysis of mediated effects

We categorized the overall effect of sleep problems on unruptured CA into direct effects as well as indirect effects mediated through mediating factors. The indirect effect of the mediator in this study was calculated using the product method, which is the indirect effect of sleep problems on unruptured CA through the mediator. The proportion of the mediator in this study was obtained from the ratio of the indirect effect to the overall effect, and the corresponding confidence intervals were obtained using the Delta method.

### 2.6 Statistical analysis

The data screening and processing involved in this study, as well as MR-related operations, were performed using R version 4.2.0. The data filtering and processing, as well as MR-related operations involved in this study, were also performed using R version 4.2.0. Additionally, MR-related operations were performed using the software packages “TwoSampleMR” and “MRPRESSO.” As there were four exposure factors in this study, Bonferroni correction was required, and the corrected *p*-value <0.0125 (0.05/4) was considered statistically significant. Finally, the MVMR analysis of the results used a *p*-value of less than 0.05 to indicate statistical significance.

## 3 Results

### 3.1 Results of hypothesis testing for MR

After screening, we selected the IVs associated with sleep problems, selecting 17, 28, 16, and 47 SNPs for snoring, insomnia, narcolepsy, and napping during the day, respectively (Supplementary Tables S1–S4). To assess the correlation between these SNPs and the corresponding exposure factors, we calculated F-statistic values for each of the four exposures. The results were 15.9, 14.5, 17.4, and 18.2 for snoring, insomnia, narcolepsy, and napping during the day, respectively, all exceeding 10, indicating a strong correlation between the selected IVs and the exposure factors.

### 3.2 Sensitivity validation results

**FIGURE 2:**
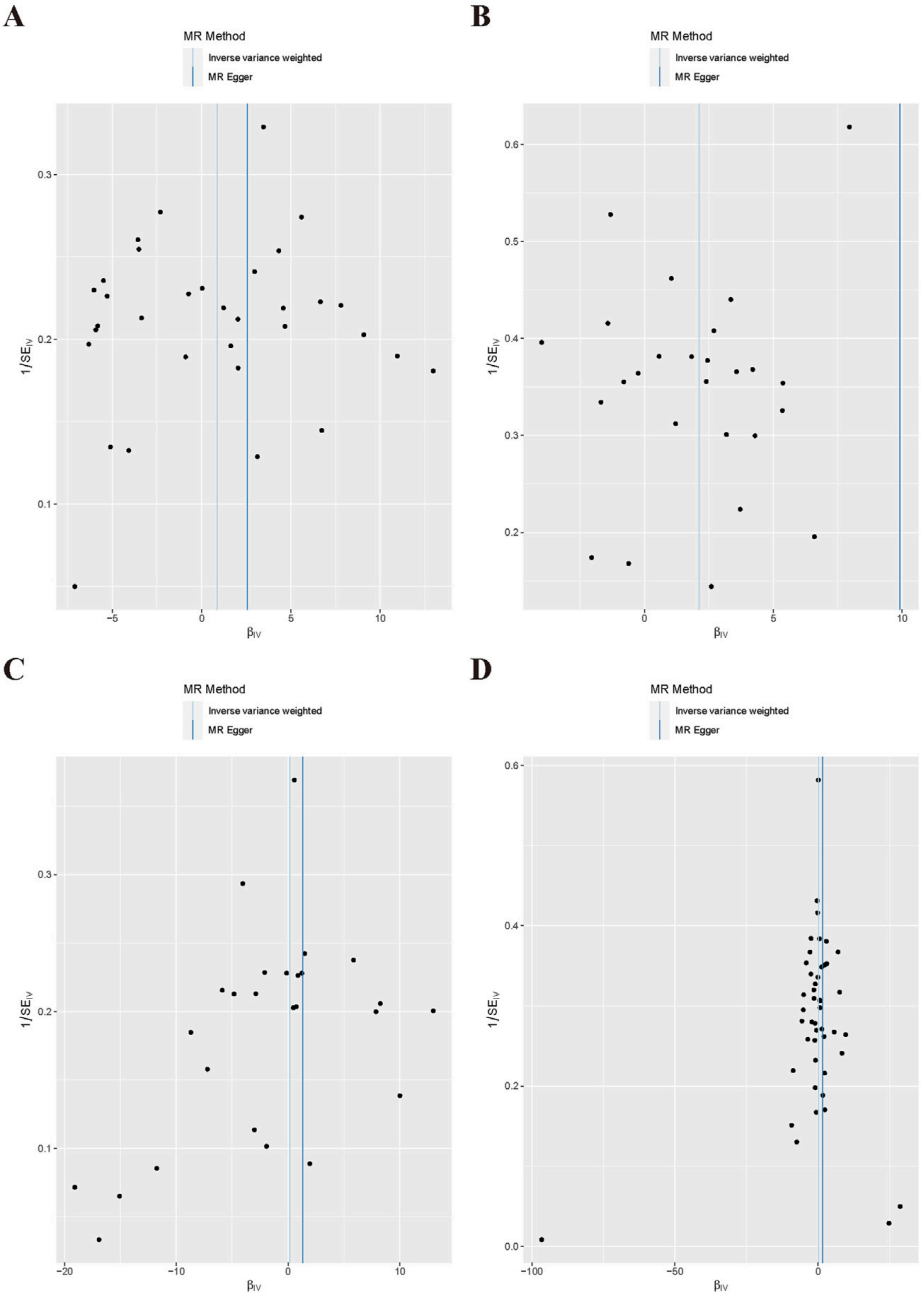
Scatter plot of MR Analysis. **(A)** Snoring; **(B)** Insomnia; **(C)** Narcolepsy; **(D)** Napping during the day.

**FIGURE 3:**
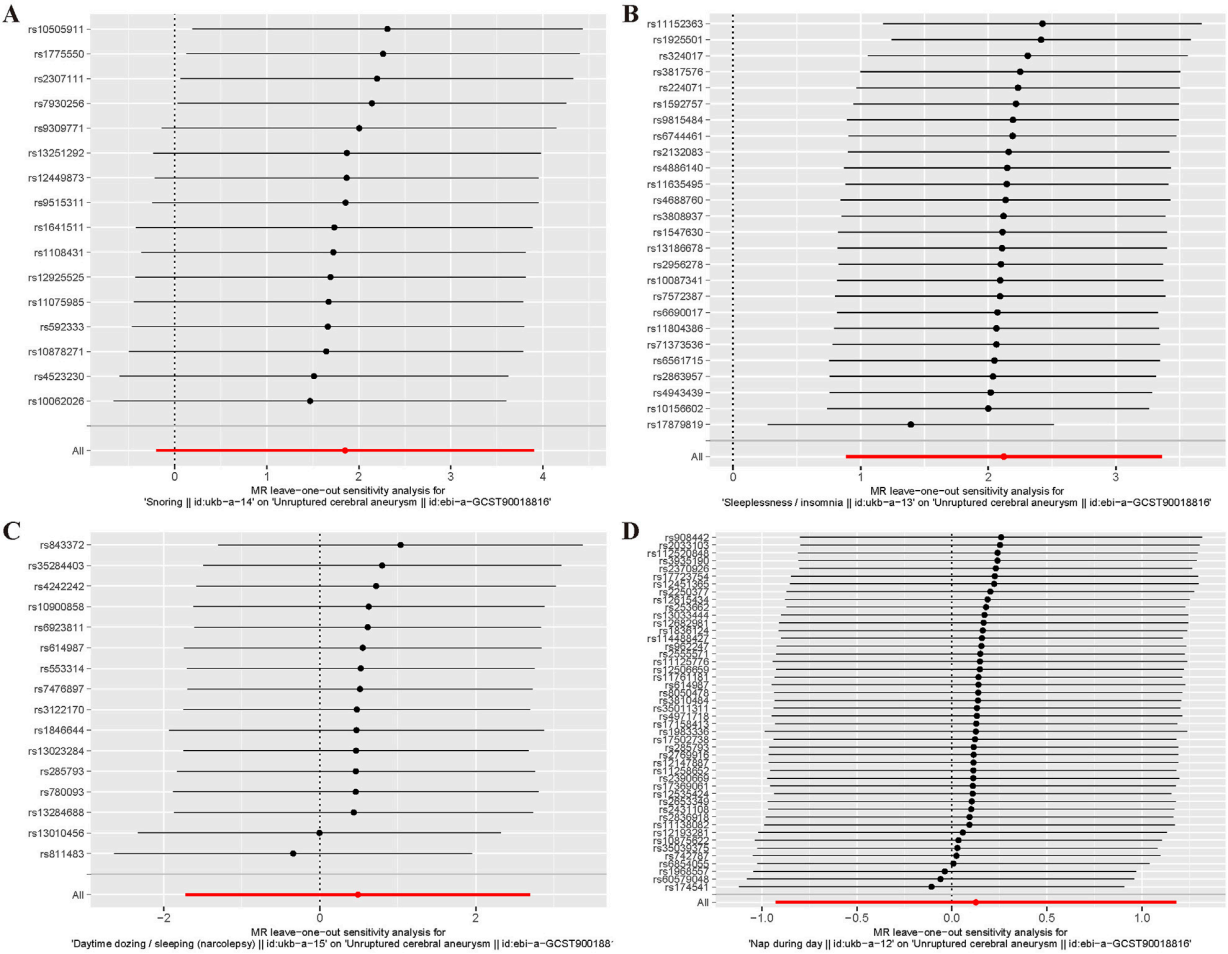
Left - one method diagram of MR Analysis. **(A)** Snoring; **(B)** Insomnia; **(C)**Narcolepsy; **(D)** Napping during the day.

For the heterogeneity test, we calculated the heterogeneity of both the IVW and MR-Egger methods, and the results of Cochrane’s Q test suggested that the *p*-values of all tests were greater than 0.05. This indicated that there was presently no heterogeneity in the IVs selected for this study (Table 2). In addition, as demonstrated in Figure 2, we observed the distribution of SNPs through funnel plots to determine the heterogeneity of these IVs. We found that, especially in Figures 2A, B, the distribution of these SNPs was more symmetrical. To further test the effect of horizontal pleiotropy on the results of this study, we performed the test by the leave-one-out method (Figure 3), and we found that there was no significant horizontal pleiotropy for the individual effects of the selected SNPs.

**TABLE 2:** The estimations of heterogeneity and horizontal pleiotropy for MR results.

Exposures	Inverse variance weighted		MR-Egger		MR-PRESSO
	Q-statistic	*p*	Q-statistic	*p*	*p* for global test
Snoring	12.598	0.633	12.569	0.561	0.273
Insomnia	34.275	0.102	28.167	0.253	0.114
Narcolepsy	13.169	0.589	10.623	0.715	0.547
Nap during day	53.303	0.185	52.826	0.170	0.123

### 3.3 MR analysis results

**FIGURE 4:**
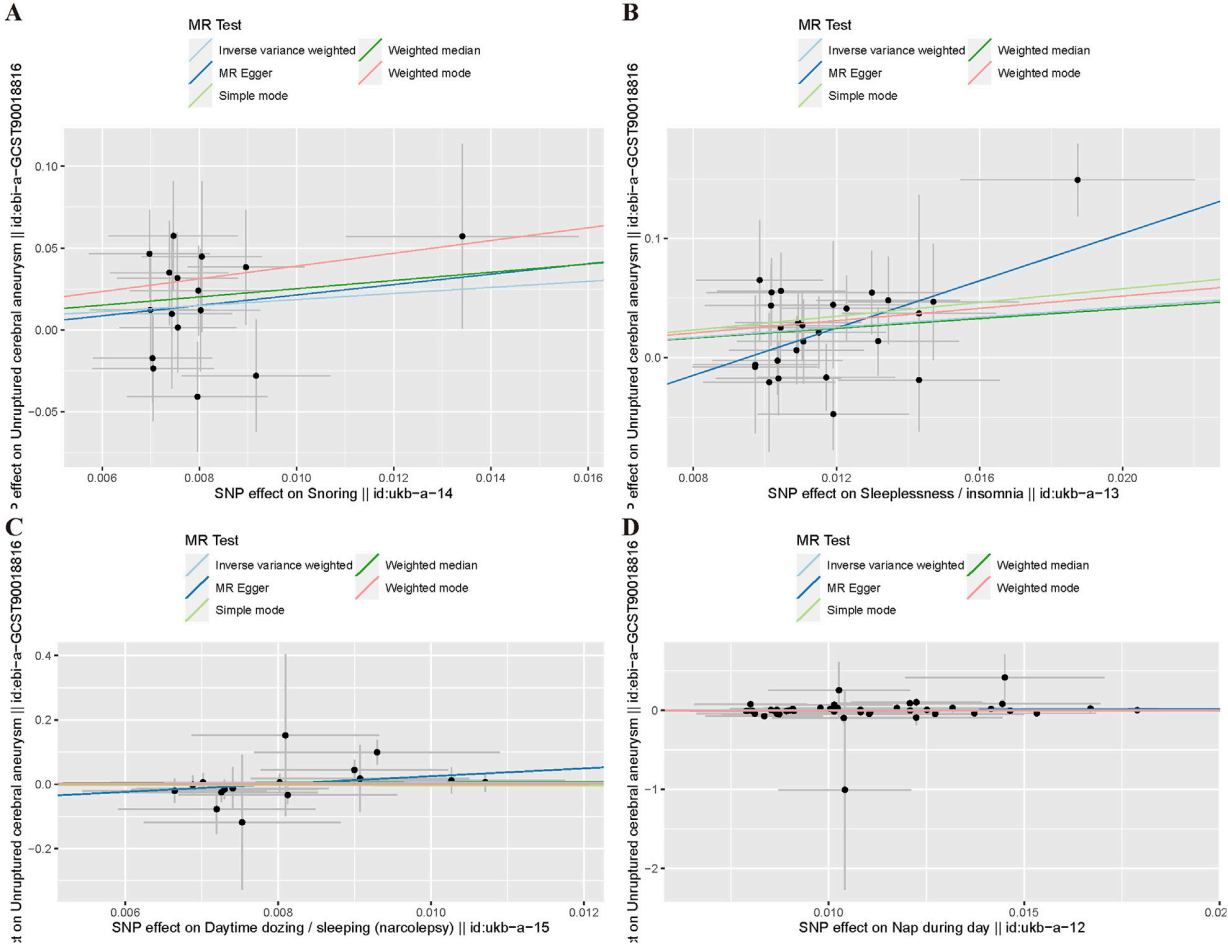
Funnel plot of MR Analysis. **(A)** Snoring; **(B)** Insomnia; **(C)** Narcolepsy; **(D)** Napping during the day.

Table 3 presents the results of the six MR analysis methods, along with the corresponding risk ratios and confidence intervals (CI). From the results in Table 3 and Figure 4, we found a significant causal effect between insomnia and CA, which was verified in the IVW [odds ratio (OR) = 8.35, 95% CI = 2.422–28.791, *p* = 7.772e-04]. However, for the other exposure factors, no causal relationship with CA was found.

**TABLE 3:** Two-sample Mendelian randomization estimations showing the effects of sleep problems on the risk of CA.

Exposures	Inverse variance weighted		Weighted median		MR Egger	
	OR (95% CI)	*p*	OR (95% CI)	*p*	OR (95% CI)	*p*
Snoring	6.367 (4.428e-06-1.312e+08)	0.077	12.361 (7.640e-01-2.000e+02)	0.077	24.1067 (4.428e-06-1.312e+08)	0.694
Insomnia	8.350 (2.422-2.879e+01)	**7.772e-04**	7.733 (1.524-3.923e+01)	0.013	2.042e+04 (2.275e+01-1.835e+07)	**8.626 e-03**
Narcolepsy	1.631 (1.794e-01-1.483e+01)	0.664	1.844 (9.575e-02-3.550e+01)	0.685	1.939e+05 (9.560e-02-3.931e+11)	0.123
Nap during day	1.135 (3.959e-01-3.252)	0.814	9.263e-01 (2.191e-01-3.916)	0.917	5.126 (4.198e-02-6.259e+02)	0.508

Exposures	Simple mode		Weighted mode		MR-PRESSO	
	OR (95% CI)	*p*	OR (95% CI)	*p*	OR (95% CI)	*p*
Snoring	49.325 (2.853e-01-8.528e+03)	0.159	49.325 (2.832e-01-8.592e+03)	0.159	2.929 (3.466e-01-2.475e+01)	0.338
Insomnia	1.802e+01 (7.159e-01-4.538e+02)	0.091	1.324e+01 (5.373e-01-3.262e+02)	0.127	7.878 (2.430-2.554e+01)	**1.91e-03**
Narcolepsy	7.054e-01 (8.504e-03-5.851e+01)	0.879	1.120 (2.590e-01-4.484e+01)	0.954	1.631 (2.061e-01-1.290e+01)	0.547
Nap during day	7.550e-01 (5.437e-02-1.048e+01)	0.835	7.550e-01 (9.152e-02-6.228)	0.795	1.309 (4.493e-01-3.813)	0.624

Bold values indicate significant P-values.

### 3.4 Analysis of mediation effects

Next, to determine if a causal relationship exists between insomnia and CA disease, we used MVMR to analyze potential mediators. Specifically, we investigated whether hypertension, smoking (not clearly classified, refers to the occurrence of smoking behavior), and obesity act as confounders in the insomnia-CA relationship. The MVMR results, as shown in Table 4, identified only hypertension as a significant confounder, whereas smoking and obesity were not. Consequently, we conducted a two-sample MR analysis of insomnia and hypertension to explore if hypertension could be a mediator between insomnia and CA. This analysis indicated that hypertension mediates the relationship between insomnia and CA (OR = 1.933, 95% CI = 1.011–3.695, *p* = 0.046), as detailed in Supplementary Tables S5, S6 and Supplementary Figure S1. The mediation effect of hypertension accounted for 52.538% in the presence of insomnia.

**TABLE 4:** MVMR results for confounding factor analysis.

Exposures	Outcome	OR	95% CI	*p*-value
Hypertension	Unruptured cerebral aneurysm	1.562	1.304–1.870	**1.289e-06**
Insomnia	Unruptured cerebral aneurysm	5.430	1.147–25.692	**3.285e-02**
Smoking	Unruptured cerebral aneurysm	0.889	0.682–1.158	0.383
Insomnia	Unruptured cerebral aneurysm	3.812	1.365–10.645	**0.011**
Obesity	Unruptured cerebral aneurysm	1.069	0.911–1.255	0.413
Insomnia	Unruptured cerebral aneurysm	3.326	1.136–9.739	**0.028**

Bold values indicate significant P-values.

## 4 Discussion

To our knowledge, this study was the first to analyze the causal effect relationship between sleep-related problems and CA by a two-sample MR analysis method. We ultimately determined insomnia as a risk factor for CA, meeting the Bonferroni correction criteria. Other potential factors, including snoring, daytime napping, and insomnia, did not show a causal link with CA. Insomnia’s contribution as a risk factor for CA, with hypertension as a mediator, was established at 52.538%. Moreover, the sensitivity analysis, which assessed heterogeneity and horizontal pleiotropy, verified the reliability of our findings. Cerebral aneurysms, particularly brain aneurysms, have long been a covert threat due to their non-specific symptoms and prevalence in about 5% of the population. The grave consequences of a CA rupture underscore the urgency of investigating CA growth and rupture risks. A prior Japanese study involving 5,720 patients indicated a significant correlation between the risk of CA rupture and factors like its size, location, and shape (Investigators et al., 2012). Another meta-analysis consolidated multiple prospective studies that assessed the risk of aneurysms and pinpointed age over 70 years, hypertension, and the aneurysm’s size and location as predictors of rupture risk (Foreman et al., 2018; Greving et al., 2014). In particular, smoking and hypertension have been indicated as having a strong correlation with the development and rupture of CA by many studies (Connolly et al., 2012).

CA occurs mainly due to the thinning of the intima and internal elastic layer of the blood vessels in the brain, which is accelerated by atherosclerosis and leads to local deformation and bulging when the pressure increases (Lin et al., 2015). When the vessel wall is damaged, a cascade of inflammatory reactions is triggered, which leads to the breakdown of the smooth muscle cells in the mesothelium, the destruction of the internal elastic layer, and the infiltration of peripheral cells such as macrophages into the vessel wall, which destroys the tight junctions between the cells and further increases the permeability of the vessel wall, resulting in the gradual formation of the aneurysm (Meng et al., 2014; Meng et al., 2007). Once the aneurysm sac forms, it initiates hemodynamic changes, and its ongoing enlargement escalates the wall tension. The aneurysm ruptures when this tension surpasses the rupture point (Frosen et al., 2019; Sheinberg et al., 2019).

The first MR analysis of a large sample in this study revealed that insomnia is also a risk factor for CA. Insomnia, one of the most common sleep disorders, has a prevalence of up to 30% in the population (Winiger et al., 2020; Li et al., 2022). Moreover, the occurrence of insomnia can lead to damage to both physical and mental health, including decreased concentration, frequent fatigue, headaches, gastrointestinal reactions, and emotional irritability (Appleton et al., 2022). In addition, many studies have shown that insomnia may trigger the development of hypertension, cardiovascular disease, and even cancer (Appleton et al., 2022; Liu et al., 2020). The mechanisms by which insomnia causes cardiovascular disease are not yet fully understood; however, some relevant studies suggest a possible association with abnormal regulation of the hypothalamic-pituitary axis (Grandner et al., 2016), increased sympathetic nervous system activity (Parthasarathy et al., 2015), systemic inflammatory responses, and increased atherosclerosis (King et al., 2008). Furthermore, studies have shown that patients with insomnia have an abnormally active sympathetic nervous system, which leads to changes in hormone levels in the body, mainly in norepinephrine, ultimately leading to arrhythmias and coronary heart disease (Zhang et al., 2011; Johansson et al., 2011). Other prospective studies have shown that insomnia is associated with coronary heart disease, risk of recurrence of acute coronary syndromes, death, and an increased incidence of acute myocardial infarction (Laugsand et al., 2011; Coryell et al., 2013). Similarly, the findings of this study illustrate a risk association between insomnia and coronary artery disease, potentially linked to insomnia-induced hypertension. Indeed, cross-sectional studies have shown a correlation between insomnia and hypertension, suggesting that decreased sleep duration escalates hypertension risk (Phillips et al., 2009; Bathgate et al., 2016). Additionally, people who sleep less than 6 h per night exhibit a significantly higher risk of hypertension (by more than threefold) and a more than twofold increase in the risk of being treated with medication than do those who do not (Bathgate et al., 2016; Kalmbach et al., 2016).

In addition, the increased risk of CA due to insomnia may be related to its triggering of inflammatory responses and endocrine dysregulation. Inflammation occurs as an important mechanism leading to vascular disease (Alfaddagh et al., 2020), especially when the organism triggers an inflammatory response that leads to the release of large amounts of inflammatory factors, including chemokines, growth factors, and adhesion factors (Carrizales-Sepulveda et al., 2018). Studies have shown that when sleep is limited to 4 h, the transcription levels of some proinflammatory factors, such as interleukin-6 (IL-6) and tumor necrosis factor, are substantially increased in immune cells (Knutson et al., 2009). In addition, since IL-6 secretion has a circadian rhythm, less sleep will lead to increased IL-6 secretion during the day and increased blood pressure (Vgontzas et al., 1999; Gangwisch et al., 2006). When insomnia symptoms persist for a period of time, the inflammatory response in the body is aggravated, resulting in the release of more inflammatory factors, among which C-reactive protein (CRP), IL-1, IL-6 and tumor necrosis factor α (TNF-α) lead to vascular endothelial cell dysfunction and endothelium-dependent vasodilation function is reduced. As a result, vascular permeability and oxidative stress response become more intense, and these changes further aggravate vascular structural and functional abnormalities. At the same time, hypertension will also lead to increased inflammation, and the interaction between CRP, IL-1, IL-6 and TNF-α and blood pressure will eventually lead to an increased risk of CA. Moreover, the expression of C-reactive protein has been observed to increase with reduced sleep duration (Ferrie et al., 2013) and remains high even after sleep duration is restored. This trend is particularly evident in women (Miller et al., 2009). Other mechanisms include damage to the vascular endothelium due to oxidative stress (Munzel et al., 2017), altered coagulation status due to fluctuations in the expression levels of coagulation factors (Kim et al., 2010), and imbalance in the levels of insulin and leptin secretion due to endocrine disorders (Cassidy et al., 2016). In summary, insomnia affects the vascular system of the body through a variety of direct and indirect mechanisms, increasing the risk of CA and rupture. Therefore, more attention should be paid to sleep-related issues, especially the adverse consequences of insomnia, as actively improving sleep quality may better prevent the occurrence of CA.

However, this study had some limitations. First, the MR analysis minimized the interference of confounding factors and the effect of reverse causality; however, it was still difficult to eliminate the effect of pleiotropy. Furthermore, due to limitations in the GWAS database, essential patient information such as age, sex, family history, and other relevant details was unavailable, leading to certain drawbacks. As the number of databases meeting IVs requirements is small, OR values and 95% CI are too large during the analysis. For example, the CI corresponding to snoring is too large. More data should be added for further analysis. Additionally, to minimize ethnicity bias, the study utilized datasets predominantly from individuals of European descent. Although this strategy reduces bias, it limits the study’s applicability across diverse populations, necessitating further research involving varied ethnic groups. In the mediation analysis, hypertension was identified as a factor potentially mediating the link between insomnia and CA. In fact, there are many common factors between insomnia and hypertension, such as hormone levels, inflammation levels and related drug use, which may have an impact on CA. Due to the limitation of information collected in the database, we cannot know other follow-up information of patients with insomnia or hypertension for the time being. However, additional research is needed to fully understand insomnia’s role in CA. Lastly, while no causal link was established between snoring, sleepiness, and sleep problems, the association of these common sleep issues with various physical disorders in existing literature suggests the need for further comprehensive studies with larger sample sizes.

## 5 Conclusion

In summary, our study predicted the causal relationship between sleep-related issues and CA using genetic mutation data. We are the first, to our knowledge, to establish a causal link between insomnia and CA, identifying insomnia as a potential risk factor. Additionally, hypertension was found to mediate 52.538% of the relationship between insomnia and CA. Hence, increased attention to sleep problems is warranted, as mitigating hypertension could reduce the risk of CA.

## Data Availability

The original contributions presented in the study are included in the article/Supplementary Material, further inquiries can be directed to the corresponding author.

## Abbreviations

CA, Cerebral aneurysm; MR, Mendelian randomization; MVMR, multivariate MR; IV, Instrumental variable; LD, Linkage disequilibrium; SNPs, Single nucleotide polymorphisms; OR, Odds ratio; CI, Confidence interval; GWAS, Genome-wide association study; IL-6, interleukin-6; PRESSO, pleiotropy residual sum and outlier.
