# Supplementary material for: The impact of sleep problems on cerebral aneurysm risk is mediated by hypertension: a mediated Mendelian randomization study
Source: Front Genet. 2024 Oct 11;15:1434189. doi: 10.3389/fgene.2024.1434189 (PMC11502348; doi:10.3389/fgene.2024.1434189)
Supplement: Supplementary file 1 [file Table1.DOCX]

Supplementary Material

The impact of sleep problems on cerebral aneurysm risk is mediated by hypertension: A mediated Mendelian randomization study

Xiaofei Yan ^1^, Hongwu Li ^2*^

^1^ Department of Pathology, The Quzhou Affiliated Hospital of Wenzhou Medical University, Quzhou People’s Hospital, Quzhou, Zhejiang, China

^2^ Department of Neurosurgery, The Quzhou Affiliated Hospital of Wenzhou Medical University, Quzhou People’s Hospital, Quzhou, Zhejiang, China

*** Correspondence:**Hongwu Li

[573721171@qq.com](mailto:573721171@qq.com)

# Supplementary Figures


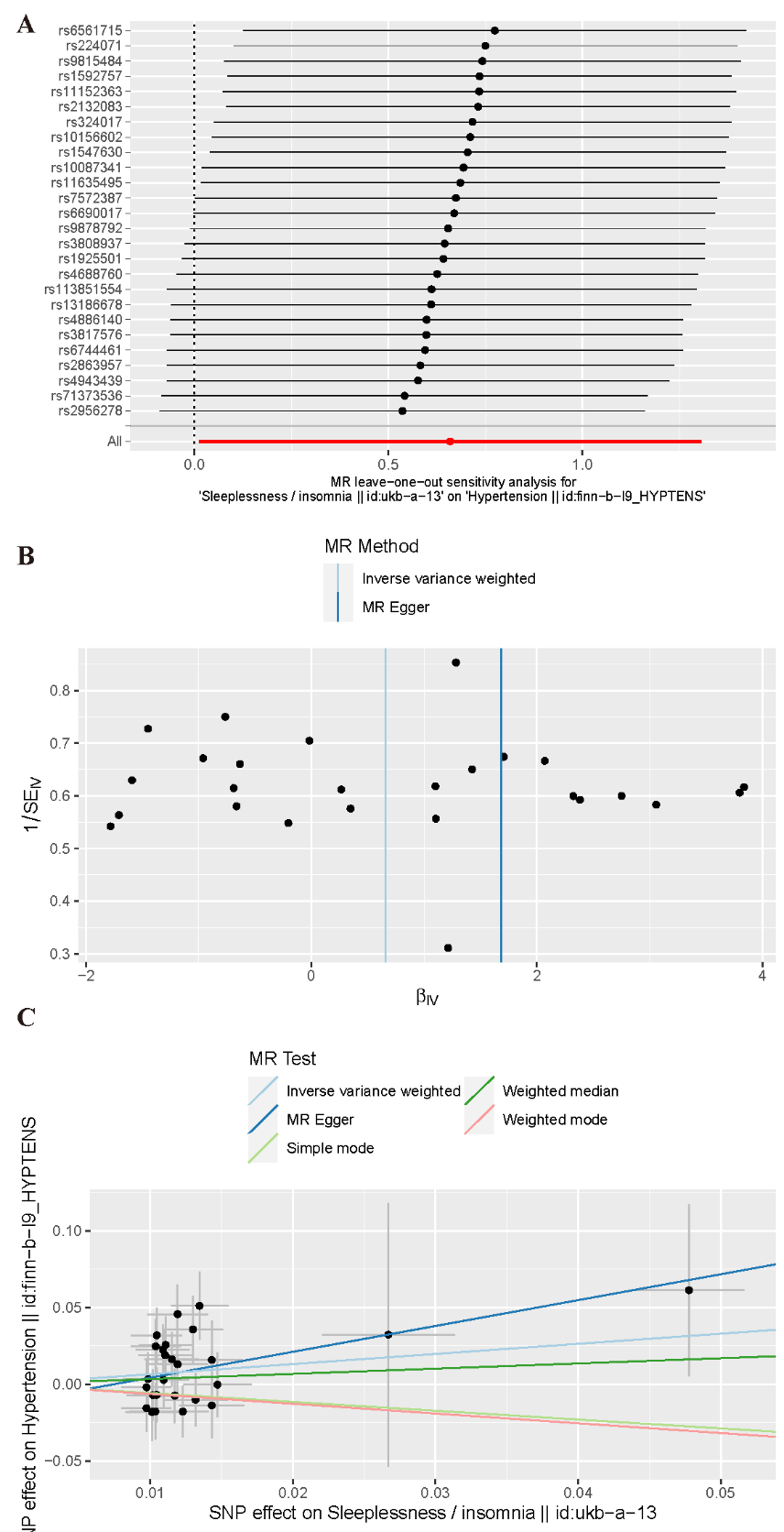


**Supplementary Figure 1: (A)** Insomnia and hypertension in two-sample of MR with leave-one method results. **(B)** Funnel plots of two-sample MR for insomnia and hypertension. **(C)** Scatterplot of two-sample MR for insomnia and hypertension.

# Supplementary Tables

**Supplementary Table 1.** Harmonized dataset of Mendelian randomization for the effect of snoring on CA.

| **SNP** | **Effect allele** | **Other allele** | **Chr** | **Exposure** | | | **Outcome** | | |
| --- | --- | --- | --- | --- | --- | --- | --- | --- | --- |
|  |  |  |  | ***β*** | **SE** | ***p*** | ***β*** | **SE** | ***p*** |
| **rs10062026** | A | G | 5 | 0.006976 | 0.001246 | 2.14E-08 | 0.046453 | 0.026942 | 0.08467 |
| **rs10505911** | A | C | 12 | -0.00796 | 0.001446 | 3.72E-08 | 0.040809 | 0.033508 | 0.223267 |
| **rs10878271** | C | T | 12 | 0.007551 | 0.001244 | 1.28E-09 | 0.03166 | 0.027825 | 0.255184 |
| **rs11041980** | A | T | 11 | 0.007899 | 0.001205 | 5.53E-11 | -0.05634 | 0.026865 | 0.035993 |
| **rs11075985** | A | C | 16 | -0.00738 | 0.001212 | 1.11E-09 | -0.03493 | 0.031867 | 0.273067 |
| **rs1108431** | T | C | 16 | -0.00805 | 0.001237 | 7.61E-11 | -0.04476 | 0.046045 | 0.33095 |
| **rs12449873** | C | G | 17 | 0.007435 | 0.001235 | 1.74E-09 | 0.009783 | 0.045704 | 0.830511 |
| **rs12925525** | T | G | 16 | -0.01341 | 0.002392 | 2.07E-08 | -0.05722 | 0.056439 | 0.310668 |
| **rs13251292** | G | A | 8 | -0.00803 | 0.001222 | 4.97E-11 | -0.01195 | 0.037448 | 0.749639 |
| **rs1641511** | A | G | 17 | 0.007981 | 0.001413 | 1.62E-08 | 0.024014 | 0.027502 | 0.38256 |
| **rs1775550** | A | G | 10 | 0.009168 | 0.001533 | 2.21E-09 | -0.02802 | 0.03446 | 0.416141 |
| **rs2307111** | C | T | 5 | 0.007038 | 0.001226 | 9.52E-09 | -0.01729 | 0.026996 | 0.521968 |
| **rs4523230** | T | A | 8 | -0.00747 | 0.001329 | 1.92E-08 | -0.05744 | 0.033399 | 0.085456 |
| **rs592333** | G | A | 13 | -0.00896 | 0.001204 | 1.01E-13 | -0.03831 | 0.034961 | 0.273128 |
| **rs7930256** | C | T | 11 | -0.00705 | 0.00125 | 1.7E-08 | 0.023595 | 0.032068 | 0.461868 |
| **rs9309771** | G | A | 3 | 0.007558 | 0.001202 | 3.22E-10 | 0.001451 | 0.027277 | 0.957588 |
| **rs9515311** | T | C | 13 | 0.006995 | 0.001238 | 1.63E-08 | 0.012173 | 0.035224 | 0.729656 |

Chr: Chromosome.

**Supplementary Table 2.** Harmonized dataset of Mendelian randomization for the effect of insomnia on CA.

| **SNP** | **Effect allele** | **Other allele** | **Chr** | **Exposure** | | | **Outcome** | | |
| --- | --- | --- | --- | --- | --- | --- | --- | --- | --- |
|  |  |  |  | ***β*** | **SE** | ***p*** | ***β*** | **SE** | ***p*** |
| **rs10087341** | C | T | 8 | 0.014712 | 0.002392 | 7.74E-10 | -0.00027 | 0.020876 | 0.989846 |
| **rs10156602** | G | A | 9 | -0.01019 | 0.001823 | 2.32E-08 | 0.007023 | 0.016573 | 0.671749 |
| **rs10280045** | G | C | 7 | 0.009726 | 0.001772 | 4.04E-08 | 0.015936 | 0.017156 | 0.352943 |
| **rs11152363** | A | G | 18 | 0.014311 | 0.002263 | 2.54E-10 | -0.01376 | 0.021314 | 0.518636 |
| **rs113851554** | T | G | 2 | 0.047777 | 0.00389 | 1.16E-34 | 0.061281 | 0.056008 | 0.273896 |
| **rs11635495** | C | T | 15 | 0.009731 | 0.001742 | 2.34E-08 | -0.00199 | 0.017753 | 0.91083 |
| **rs13186678** | T | C | 5 | 0.011048 | 0.001888 | 4.91E-09 | 0.018866 | 0.01639 | 0.249723 |
| **rs1547630** | A | G | 13 | 0.010442 | 0.001833 | 1.23E-08 | -0.00694 | 0.017997 | 0.699966 |
| **rs1592757** | C | G | 5 | 0.010353 | 0.001818 | 1.24E-08 | -0.01768 | 0.018376 | 0.335976 |
| **rs1925501** | A | T | 16 | -0.01191 | 0.002106 | 1.54E-08 | -0.01311 | 0.019271 | 0.4964 |
| **rs2132083** | C | T | 4 | -0.01012 | 0.001844 | 4.04E-08 | 0.018037 | 0.018664 | 0.333837 |
| **rs224071** | A | G | 10 | 0.009726 | 0.001749 | 2.67E-08 | -0.01548 | 0.01545 | 0.316333 |
| **rs2644128** | G | C | 1 | 0.010901 | 0.001751 | 4.82E-10 | -0.0098 | 0.016675 | 0.5566 |
| **rs2863957** | A | C | 2 | -0.01299 | 0.002104 | 6.67E-10 | -0.03574 | 0.021658 | 0.098864 |
| **rs2956278** | G | A | 12 | 0.01191 | 0.002123 | 2.03E-08 | 0.045701 | 0.019317 | 0.017989 |
| **rs324017** | C | A | 12 | -0.01172 | 0.001912 | 8.83E-10 | 0.007432 | 0.017743 | 0.675288 |
| **rs3808937** | T | C | 10 | -0.01431 | 0.002146 | 2.59E-11 | -0.01579 | 0.025703 | 0.53898 |
| **rs3817576** | G | A | 7 | -0.01038 | 0.001747 | 2.81E-09 | -0.02472 | 0.01752 | 0.158277 |
| **rs4688760** | T | C | 3 | 0.011506 | 0.001886 | 1.06E-09 | 0.0164 | 0.017693 | 0.353989 |
| **rs4886140** | G | A | 13 | 0.011077 | 0.001862 | 2.69E-09 | 0.02573 | 0.018478 | 0.163793 |
| **rs4943439** | T | C | 13 | 0.010451 | 0.001785 | 4.75E-09 | 0.031967 | 0.017924 | 0.074508 |
| **rs6561715** | A | T | 13 | -0.01228 | 0.001807 | 1.06E-11 | 0.017808 | 0.016887 | 0.291648 |
| **rs6690017** | G | T | 1 | -0.00985 | 0.001771 | 2.66E-08 | -0.00343 | 0.01711 | 0.841334 |
| **rs6744461** | C | A | 2 | 0.010883 | 0.001883 | 7.48E-09 | 0.022524 | 0.016333 | 0.167888 |
| **rs71373536** | A | G | 17 | 0.01345 | 0.002004 | 1.92E-11 | 0.051102 | 0.022193 | 0.021297 |
| **rs7572387** | C | A | 2 | 0.01093 | 0.001767 | 6.21E-10 | 0.002891 | 0.01786 | 0.871404 |
| **rs9815484** | G | A | 3 | 0.013168 | 0.00226 | 5.64E-09 | -0.01006 | 0.017551 | 0.566362 |
| **rs9878792** | T | G | 3 | -0.0267 | 0.004658 | 9.95E-09 | -0.03236 | 0.085825 | 0.70616 |

Chr: Chromosome.

**Supplementary Table 3.** Harmonized dataset of Mendelian randomization for the effect of narcolepsy on CA.

| **SNP** | **Effect allele** | **Other allele** | **Chr** | **Exposure** | | | **Outcome** | | |
| --- | --- | --- | --- | --- | --- | --- | --- | --- | --- |
|  |  |  |  | ***β*** | **SE** | ***p*** | ***β*** | **SE** | ***p*** |
| **rs10900858** | G | A | 5 | -0.00665 | 0.001202 | 3.18E-08 | 0.020386 | 0.038529 | 0.596723 |
| **rs13010456** | G | A | 2 | -0.009 | 0.001227 | 2.21E-13 | -0.04482 | 0.032213 | 0.164099 |
| **rs13023284** | C | T | 2 | -0.0081 | 0.001227 | 4.15E-11 | -0.15215 | 0.251981 | 0.545978 |
| **rs13284688** | C | T | 9 | 0.010268 | 0.001485 | 4.75E-12 | 0.01158 | 0.041394 | 0.779665 |
| **rs1846644** | C | T | 12 | 0.010709 | 0.001223 | 1.96E-18 | 0.006337 | 0.030622 | 0.836049 |
| **rs285793** | A | G | 8 | -0.00702 | 0.001208 | 6.33E-09 | -0.00585 | 0.029142 | 0.840831 |
| **rs3122170** | A | C | 6 | -0.00907 | 0.00143 | 2.24E-10 | -0.01786 | 0.104051 | 0.863735 |
| **rs35284403** | C | T | 7 | 0.007258 | 0.001262 | 8.83E-09 | -0.0248 | 0.030134 | 0.41052 |
| **rs4242242** | A | G | 5 | -0.0073 | 0.001217 | 2.01E-09 | 0.016145 | 0.02916 | 0.5798 |
| **rs553314** | C | T | 1 | -0.00741 | 0.001256 | 3.70E-09 | 0.012875 | 0.065798 | 0.844861 |
| **rs614987** | C | A | 6 | 0.006882 | 0.001238 | 2.71E-08 | -0.00207 | 0.029026 | 0.943016 |
| **rs6923811** | C | T | 6 | -0.0072 | 0.001289 | 2.39E-08 | 0.077423 | 0.077237 | 0.316143 |
| **rs7476897** | A | G | 10 | -0.00753 | 0.001287 | 4.87E-09 | 0.118184 | 0.209936 | 0.573468 |
| **rs780093** | C | T | 2 | -0.00802 | 0.001236 | 8.62E-11 | -0.00577 | 0.027097 | 0.831461 |
| **rs811483** | T | C | 1 | -0.00929 | 0.001608 | 7.52E-09 | -0.0993 | 0.038143 | 0.00923 |
| **rs843372** | T | C | 3 | -0.00813 | 0.001431 | 1.37E-08 | 0.033386 | 0.028076 | 0.234387 |

Chr: Chromosome.

**Supplementary Table 4.** Harmonized dataset of Mendelian randomization for the effect of nap during day on CA.

| **SNP** | **Effect allele** | **Other allele** | **Chr** | **Exposure** | | | **Outcome** | | |
| --- | --- | --- | --- | --- | --- | --- | --- | --- | --- |
|  |  |  |  | ***β*** | **SE** | ***p*** | ***β*** | **SE** | ***p*** |
| **rs10875622** | A | G | 5 | 0.009793 | 0.001458 | 1.89E-11 | 0.030046 | 0.02776 | 0.27909 |
| **rs11125776** | G | T | 2 | -0.01464 | 0.002049 | 8.96E-13 | 0.004624 | 0.033946 | 0.891654 |
| **rs11138082** | C | T | 9 | 0.014158 | 0.001785 | 2.19E-15 | 0.017561 | 0.040614 | 0.665459 |
| **rs112520848** | C | G | 17 | 0.008114 | 0.001486 | 4.77E-08 | -0.04164 | 0.027497 | 0.12997 |
| **rs11258652** | A | C | 10 | -0.01004 | 0.001693 | 3.01E-09 | -0.01699 | 0.053284 | 0.749856 |
| **rs114488427** | T | C | 16 | 0.01224 | 0.002207 | 2.92E-08 | -0.09119 | 0.094054 | 0.332263 |
| **rs11761181** | C | T | 7 | 0.010815 | 0.001716 | 2.92E-10 | -0.00944 | 0.046588 | 0.839394 |
| **rs12147887** | T | C | 14 | 0.010814 | 0.001856 | 5.65E-09 | 0.007383 | 0.035343 | 0.834523 |
| **rs12193281** | C | T | 6 | -0.00979 | 0.001531 | 1.58E-10 | -0.02317 | 0.027894 | 0.406082 |
| **rs12451365** | C | T | 17 | 0.011061 | 0.001784 | 5.6E-10 | -0.02702 | 0.028777 | 0.347807 |
| **rs12506659** | T | C | 4 | -0.00892 | 0.001447 | 7.01E-10 | 0.010083 | 0.034692 | 0.771335 |
| **rs12535424** | A | G | 7 | -0.01451 | 0.00255 | 1.28E-08 | -0.41633 | 0.292454 | 0.154573 |
| **rs12615434** | T | C | 2 | 0.012725 | 0.002273 | 2.16E-08 | -0.04531 | 0.049246 | 0.357505 |
| **rs12682981** | G | A | 9 | -0.01014 | 0.00166 | 9.91E-10 | 0.014711 | 0.031693 | 0.642526 |
| **rs13033444** | G | A | 2 | 0.01082 | 0.001603 | 1.47E-11 | -0.02361 | 0.038646 | 0.541198 |
| **rs17158413** | A | G | 15 | 0.010418 | 0.001694 | 7.71E-10 | -1.00614 | 1.26448 | 0.42621 |
| **rs17369061** | C | T | 1 | -0.01175 | 0.002151 | 4.77E-08 | -0.0284 | 0.069018 | 0.680693 |
| **rs174541** | C | T | 11 | 0.010144 | 0.001498 | 1.28E-11 | 0.071076 | 0.027615 | 0.010059 |
| **rs17502738** | C | T | 9 | -0.01026 | 0.001816 | 1.58E-08 | -0.25455 | 0.356795 | 0.475577 |
| **rs17723754** | T | C | 18 | -0.01373 | 0.001458 | 4.75E-21 | 0.038275 | 0.037393 | 0.306031 |
| **rs1836124** | T | C | 11 | -0.00864 | 0.001572 | 3.96E-08 | 0.012043 | 0.027897 | 0.665967 |
| **rs1843815** | T | A | 1 | 0.008094 | 0.001447 | 2.23E-08 | 0.057171 | 0.027557 | 0.038015 |
| **rs1968557** | T | C | 5 | -0.008 | 0.001441 | 2.82E-08 | -0.07704 | 0.030299 | 0.011006 |
| **rs1983336** | A | G | 12 | 0.017891 | 0.001471 | 5.08E-34 | 0.002282 | 0.030739 | 0.940831 |
| **rs2033103** | T | C | 18 | 0.00868 | 0.001447 | 2.01E-09 | -0.04361 | 0.027659 | 0.114864 |
| **rs2250377** | G | A | 1 | -0.01533 | 0.00152 | 6.82E-24 | 0.038095 | 0.045085 | 0.398126 |
| **rs2370926** | C | T | 14 | -0.00836 | 0.001498 | 2.45E-08 | 0.072826 | 0.038106 | 0.055986 |
| **rs2390669** | C | A | 2 | -0.01251 | 0.00216 | 6.91E-09 | -0.00626 | 0.0326 | 0.847657 |
| **rs2431108** | C | T | 5 | 0.013171 | 0.001532 | 8.1E-18 | 0.030398 | 0.060972 | 0.618086 |
| **rs253662** | C | T | 3 | 0.010388 | 0.001834 | 1.47E-08 | -0.09616 | 0.068724 | 0.161729 |
| **rs2555571** | G | T | 8 | -0.00789 | 0.001442 | 4.43E-08 | 0.008416 | 0.028361 | 0.766662 |
| **rs2653349** | G | A | 6 | -0.0167 | 0.001749 | 1.36E-21 | -0.02232 | 0.061605 | 0.717066 |
| **rs2769916** | A | G | 13 | 0.008526 | 0.001558 | 4.45E-08 | 0.005686 | 0.027741 | 0.837591 |
| **rs2836918** | C | T | 21 | 0.009083 | 0.0016 | 1.37E-08 | 0.019264 | 0.034697 | 0.578751 |
| **rs285793** | A | G | 8 | -0.00868 | 0.001448 | 2.08E-09 | -0.00585 | 0.029142 | 0.840831 |
| **rs35011311** | T | G | 12 | -0.00896 | 0.00164 | 4.69E-08 | 0.005698 | 0.053534 | 0.915241 |
| **rs35039375** | G | A | 1 | 0.014446 | 0.002506 | 8.18E-09 | 0.081447 | 0.054011 | 0.131564 |
| **rs3810484** | G | A | 20 | -0.00806 | 0.001448 | 2.68E-08 | 0.007936 | 0.040718 | 0.845476 |
| **rs3935190** | A | G | 17 | 0.008738 | 0.001451 | 1.72E-09 | -0.04924 | 0.031077 | 0.113084 |
| **rs4971718** | T | C | 2 | 0.009003 | 0.001438 | 3.85E-10 | -0.00037 | 0.026812 | 0.9889 |
| **rs60579048** | G | T | 1 | -0.01208 | 0.001924 | 3.39E-10 | -0.09124 | 0.038091 | 0.01661 |
| **rs614987** | C | A | 6 | 0.012085 | 0.001484 | 3.82E-16 | -0.00207 | 0.029026 | 0.943016 |
| **rs6854055** | T | G | 4 | 0.012247 | 0.001662 | 1.71E-13 | 0.102531 | 0.050805 | 0.043577 |
| **rs742787** | C | T | 6 | -0.01023 | 0.00149 | 6.65E-12 | -0.02996 | 0.026867 | 0.264868 |
| **rs8050478** | A | G | 16 | -0.00794 | 0.00144 | 3.55E-08 | 0.004281 | 0.02944 | 0.88439 |
| **rs908442** | T | A | 2 | -0.01104 | 0.001467 | 5.31E-14 | 0.045224 | 0.031215 | 0.147386 |
| **rs962247** | A | G | 18 | -0.00911 | 0.001453 | 3.55E-10 | 0.008642 | 0.027838 | 0.756218 |
| **rs10875622** | A | G | 5 | 0.009793 | 0.001458 | 1.89E-11 | 0.030046 | 0.02776 | 0.27909 |

Chr: Chromosome.

**Supplementary Table 5.** The estimations of heterogeneity and horizontal pleiotropy for the effects of insomnia on the risk of hypertension.

| **Exposures** | **Inverse variance weighted** | | **MR-Egger** | | **MR-PRESSO** |
| --- | --- | --- | --- | --- | --- |
|  | **Q-statistic** | ***p*** | **Q-statistic** | ***p*** | ***p* for global test** |
| **Insomnia** | 27.873 | 0.314 | 27.213 | 0.295 | 0.375 |

**Supplementary Table 6.** Two-sample MR estimations showing the effects of insomnia on the risk of hypertension.

| **Exposures** | **Inverse variance weighted** | | **Weighted median** | | **MR Egger** | |
| --- | --- | --- | --- | --- | --- | --- |
|  | **OR (95%CI)** | ***p*** | **OR (95%CI)** | ***p*** | **OR (95%CI)** | ***p*** |
| **Insomnia** | 1.933(1.011-3.695) | **0.046** | 1.404(0.570-3.458) | 0.460 | 5.391(0.357-81.362) | 0.236 |
| **Exposures** | **Simple mode** | | **Weighted mode** | | **MR-PRESSO** | |
|  | **OR (95%CI)** | ***p*** | **OR (95%CI)** | ***p*** | **OR (95%CI)** | ***p*** |
| **Insomnia** | 0.563(0.073- 4.314) | 0.585 | 0.530(0.079- 4.314) | 0.519 | 1.972(1.011-3.466) | 0.056 |
